# The predictive value of modified-Naples prognostic score for patients with locally advanced non-small cell lung cancer undergoing surgery after neoadjuvant chemotherapy

**DOI:** 10.3389/fimmu.2026.1748521

**Published:** 2026-01-28

**Authors:** Yang Wang, Chunyan Tang, Junyuan Bing, Rebeka Dejenie, Yanfei Zhang, Fangchao Li, Xiaolong Fang, Xiaotong Chen, Min Yang, Yunxia Zang, Jingjing Li

**Affiliations:** 1 Affiliated Hospital of Shandong Second Medical University, Weifang, China; 2 Jinming Yu Academician Workstation of Oncology, Shandong Second Medical University, Shandong, China; 3 Cancer Center, University of Chicago, Chicago, IL, United States; 4 School of Medicine, University of California, Davis, Davis, CA, United States

**Keywords:** locally advanced non-small cell lung cancer, modified Naples prognostic score, Naples prognostic score, prognostic biomarkers, prognostic factors

## Abstract

**Objective:**

To evaluate the prognostic significance of the modified Naples Prognostic Score (mNPS) in patients with locally advanced non-small cell lung cancer (NSCLC) after neoadjuvant chemotherapy and surgery.

**Methods:**

We conducted 126 patients with locally advanced NSCLC who were surgically treated at the Affiliated Hospital of Shandong Second Medical University from September 2012 to April 2019. According to the albumin, cholesterol, neutrophil-to-lymphocyte ratio (NLR), and lymphocyte-to-monocyte ratio (LMR) before neoadjuvant chemotherapy, mNPS was divided into separate Groups: Group 0, Group 1, and Group 2. The Kaplan-Meier method was used to analyze survival curves according to mNPS. Univariate and multivariate Cox analyses of overall survival (OS) and progression-free survival (PFS) were then conducted.

**Results:**

Based on the mNPS system, the three Groups were defined as follows: Group 0, 20(15.9%) patients; Group 1, 85(67.5%) patients; and Group 2, 21(16.7%) patients. MNPS had a higher predictive value for OS (AUC = 0.640, P = 0.007) and PFS (AUC = 0.623, P = 0.024). Univariate analysis showed that clinical stage (P = 0.004), KPS score (P = 0.003), and surgical method (P = 0.042) were significantly correlated with OS. Clinical stage (P = 0.005), KPS score (P = 0.002), and mNPS (Group 2 vs Group 0, P = 0.002; Group 1 vs Group 0, P = 0.010) were significantly associated with PFS. Based on the positive results of univariate analyses, we performed multivariate analysis. Multivariate Cox Regression showed that clinical stage (P = 0.022), KPS score (P = 0.017), and mNPS (Group 2 vs Group 0, P = 0.008; Group 1 vs Group 0, P = 0.038) were independent prognostic factors for PFS.

**Conclusion:**

MNPS was an independent prognostic factor for PFS in patients with locally advanced non-small cell lung cancer undergoing surgery after neoadjuvant chemotherapy, but it was not an independent prognostic factor for OS. Comparatively, NPS had a higher significance in predicting the prognosis of resected locally advanced NSCLC patients receiving neoadjuvant chemotherapy and surgery.

## Introduction

In cancer statistics, lung cancer has the highest mortality rate. Non-small cell lung cancer (NSCLC) accounts for up to 85% of all lung cancers (1, 2). Early-stage NSCLC, due to the lack of obvious symptoms, misses the best treatment time and is often discovered at a locally advanced or even metastatic stage (3, 4). Patients with locally advanced NSCLC often exhibit significant tumor heterogeneity. This heterogeneity is associated with an unfavorable prognosis and a dismal five-year survival rate (5, 6). According to current lung cancer treatment guidelines, locally advanced NSCLC patients could accept surgical treatment after neoadjuvant therapy (7, 8). Therefore, there is a pressing need to stratify advanced NSCLC patients to identify robust prognostic biomarkers. Previous studies have demonstrated that single inflammatory or nutritional markers have predictive value for prognosis. However, single markers often have limitations and instability. Therefore, a comprehensive predictive index combining multiple markers may have higher predictive value (9, 10). Against this backdrop, the Naples Prognostic Score (NPS), which incorporates systemic inflammation and nutritional status, was proposed (11, 12).

Currently, NPS has been confirmed to hold independent prognostic value for cancers affecting the lung, rectum, endometrium, breast, esophagus, and pancreas (13–18). NPS has emerged as an independent and practical prognostic tool in NSCLC (19). In our previous study, NPS was further validated in a cohort of locally advanced NSCLC patients who received surgical resection after neoadjuvant chemotherapy, confirming that it served as an independent predictor for both progression in free survival (PFS) and overall survival (OS) (20). The modified Naples Prognostic Score (mNPS) was composed of the optimal cut-points of NPS reconfigured using X-tile software (21). Current studies have indicated that mNPS re-established based on relevant research data is valuable for the prognosis of oral squamous cell carcinoma and colon cancer (22, 23). Presently, there is an absence of robust evidence regarding the prognostic role of mNPS following surgical resection for locally advanced NSCLC.

Hence, our work sought to delineate mNPS and clinicopathological variables and to determine its prognostic value in locally advanced NSCLC. Furthermore, the mNPS was compared with the NPS to evaluate whether it has a higher predictive value.

## Data and methods

### Clinical data

The 126 patients included in this study were all from the Second Affiliated Hospital of Shandong Medical University (September 2012 - April 2019). Inclusion criteria: (1) Patients were over 18 years old; (2) Patients were diagnosed with NSCLC by histopathology; (3) Patients had a Karnofsky Performance Status (KPS) score of 80–100; (4) Patients had not received anti-tumor treatment before admission; (5) Peripheral blood tests had been completed one week before treatment, including albumin, cholesterol, neutrophils, monocytes, lymphocytes, and tumor markers; (6) Patients did not have any major internal medical conditions and must met the indications for chemotherapy and surgery as confirmed jointly by oncologists and thoracic surgeons; (7) The patients and their families agreed to the surgery and preoperative neoadjuvant chemotherapy, and already signed informed consent forms. Exclusion criteria: (1) Concurrent malignant tumors; (2) Previous use of antibiotics or nonsteroidal anti-inflammatory drugs; (3) Presence of chronic or active infectious diseases.

### Data collection

We have collected the following information: gender, age, smoking status, pathological type, tumor location, clinical stage, degree of differentiation, KPS score, CEA level, chemotherapy regimen, surgical procedure, postoperative complications, PFS, OS, as well as albumin, cholesterol, neutrophil, monocyte counts, and lymphocyte was measured one week before neoadjuvant chemotherapy. There were no missing data for any of the patients included in the analysis.

### Treatment methods

According to current lung Cancer treatment guidelines, treatment plans were developed for all patients receiving chemotherapy through multidisciplinary discussion and evaluation by senior oncologists. Specific chemotherapy regimens included: pemetrexed combined with platinum-based drugs, paclitaxel combined with platinum-based drugs, gemcitabine combined with platinum-based drugs, and pemetrexed or paclitaxel combined with platinum-based chemotherapy. Specific dosages were determined based on the patient’s tolerance, tumor status, and the dosage and duration of chemotherapy drugs.

Patients rested for two weeks after chemotherapy. If there were no contraindications, surgery could then proceed; if contraindications existed, the rest period was extended until surgical indications were met. The specific surgical approach depended on the patient’s lesion characteristics; thoracoscopic surgery or open thoracotomy was selected. The choice between the two surgical approaches was made after a comprehensive evaluation by at least three thoracic surgeons to ensure surgical safety and minimize patient injury.

### Follow-up

All enrolled patients underwent regular follow-up. Follow-up was primarily conducted via telephone or outpatient examinations. Follow-up assessments were scheduled at three-month intervals during the initial three-year period, transitioning to six-month intervals for the subsequent six years. Scheduled follow-up included physical examinations, imaging studies, and laboratory tests.

OS was measured from the date of initial treatment to the date of all-cause death or the last known follow-up. PFS was assessed as the time from treatment initiation to the first evidence of disease progression, including local recurrence, metastatic spread, as well as death.

### Construction of mNPS

**Figure 1:**
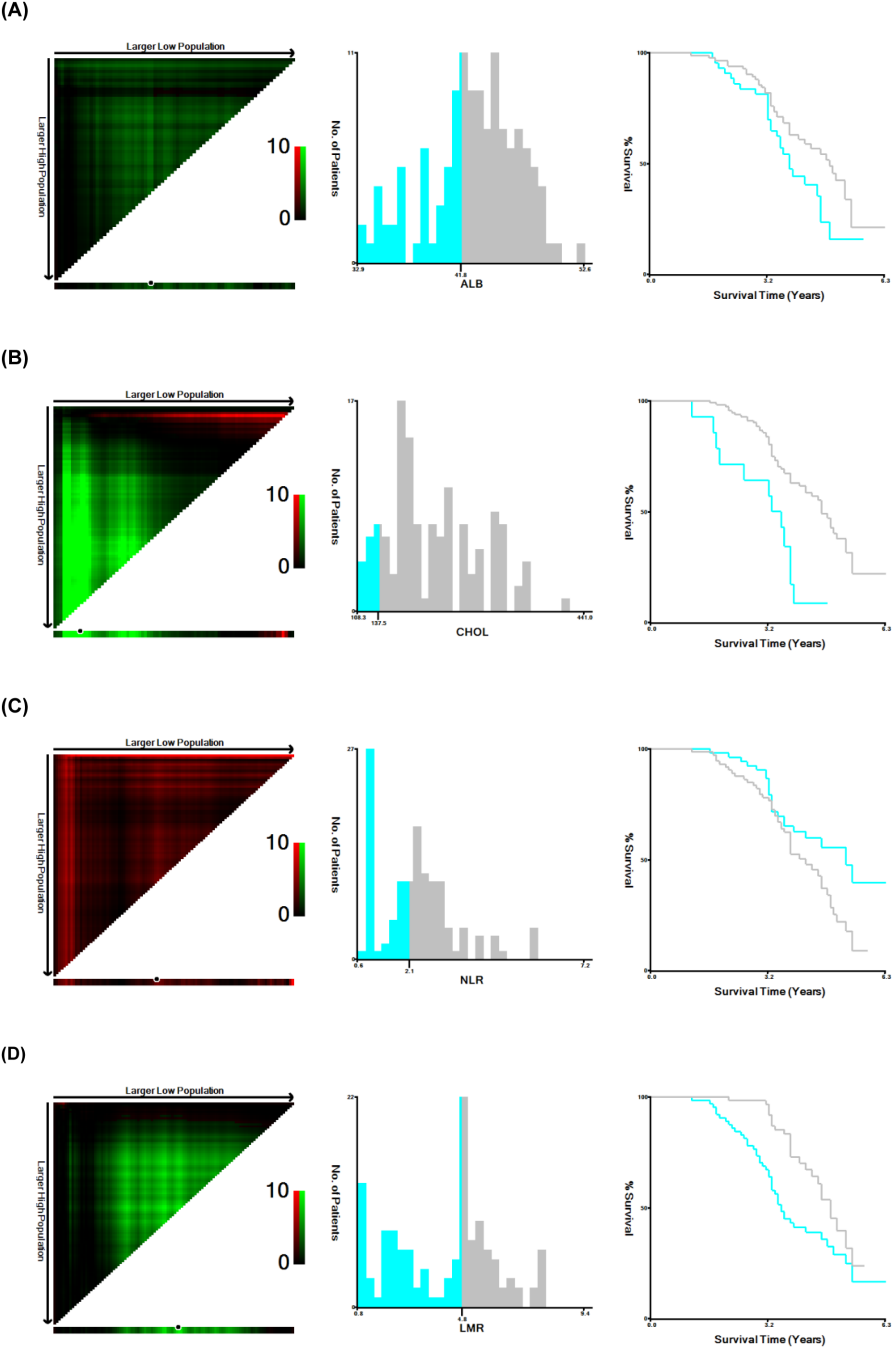
Identifying optimal cut-points of ALB, CHOL, NLR, and LMR using X-tile analysis. Optimal cut-off points for ALB, CHOL, NLR, and LMR were determined by X-tile analysis. The plots displayed the χ² log-rank values obtained by dividing the cohort into two Groups at the optimal cutoff points. Red coloration of cut points indicates an inverse correlation with survival, while green ones indicate a positive correlation. The optimal cut-points highlighted by black circles in the left panel are displayed in the histogram (middle panel), with Kaplan-Meier curves shown in the right panel. For OS, the optimal cut-points for ALB **(A)**, CHOL **(B)**, NLR **(C)**, and LMR **(D)** were 41.8, 137.5, 2.1, and 4.8, respectively.

MNPS was calculated based on cholesterol, albumin, NLR, and LMR. Data analysis was conducted with X-tile 3.6.1 (Yale University, New Haven, CT, USA), aiming at establishing cohort-specific optimal cutoffs for cholesterol, albumin, NLR, and LMR. (**Figure 1**) The modified Naples prognostic score was constructed as follows: albumin concentration >41.8 g/L = 0 points, ≤41.8 g/L = 1 point; cholesterol concentration >137.5 mg/dL = 0 points, ≤137.5 mg/dL = 1 point; NLR ≤2.1 = 0 points, >2.1 = 1 point; LMR >4.8 = 0 points, ≤4.8 = 1 point. The total score of the four indicators was the mNPS. Group 0 had an mNPS score of 0, Group 1 had scores of either1 or 2, and Group 2 had scores of 3 or 4.

### Statistical analysis

Statistical analysis was performed using IBM SPSS Statistics, Version 27.0 (SPSS Inc., Chicago, IL). The predictive accuracy of the mNPS, albumin, cholesterol, NLR, and LMR was evaluated using receiver operating characteristic (ROC) methodology. Survival outcomes across Groups were compared using the Kaplan-Meier method with comparison by the log-rank test. Univariate and multivariate Cox proportional hazards models were employed to identify candidate and delineate independent prognostic factors, respectively, with results expressed as hazard ratios (HR) and corresponding 95% confidence intervals (95% CI). Proportional hazards assumptions for all Cox regression models were verified using Schoenfeld residuals.

## Results

### Clinical characteristics

**Figure 2:**
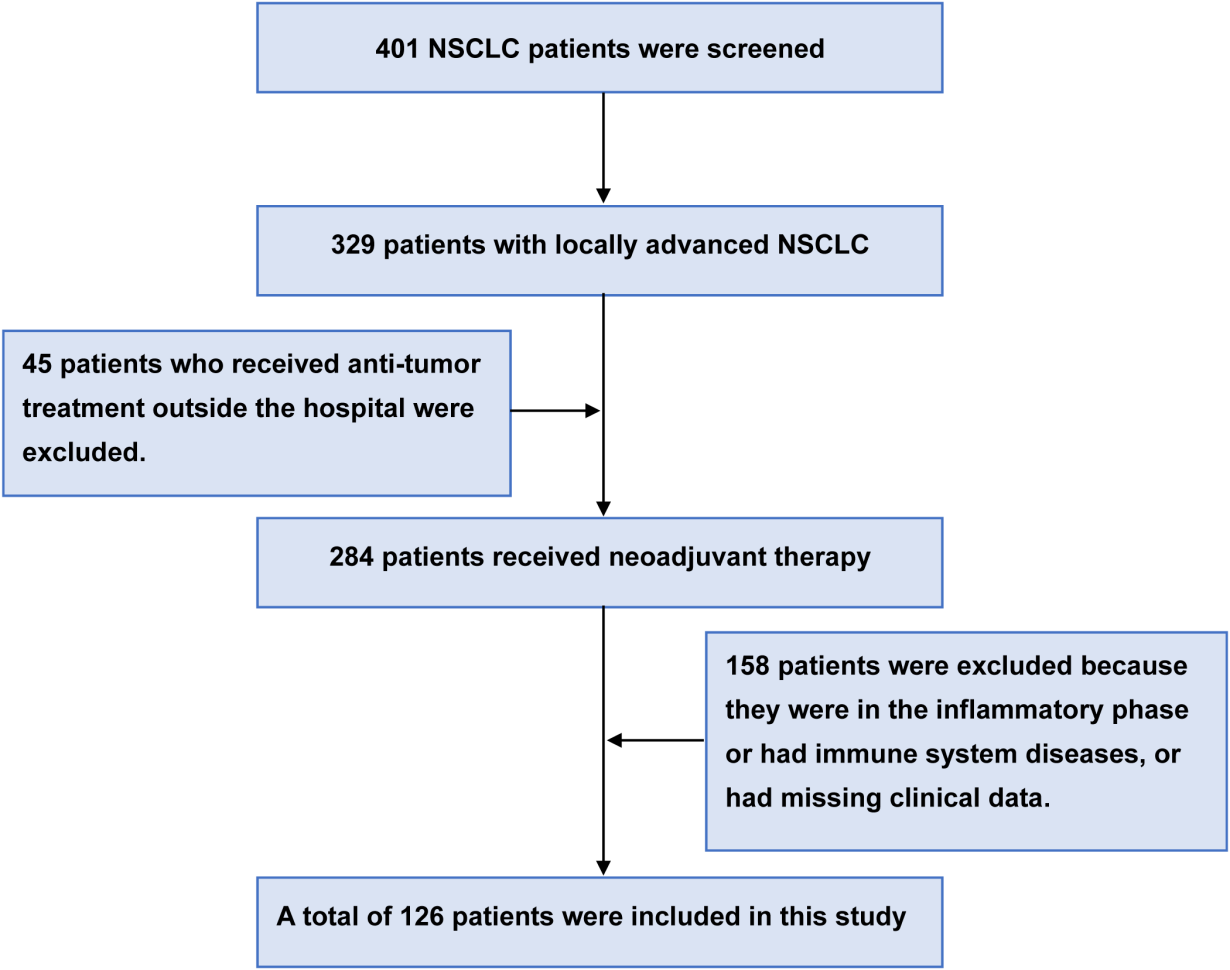
Demonstrated case screening process. A total of 401 patients with NSCLC were initially screened. After excluding 45 patients who had received prior anti-tumor therapy elsewhere and 158 patients due to inflammatory/immune diseases or insufficient clinical data, 126 were ultimately included in the study.

126 patients were included through strict inclusion and exclusion criteria. All patients received neoadjuvant chemotherapy prior to surgery and underwent regular follow-up (**Figure 2**). 66 patients (52.4%) were female, and 60 patients (47.6%) were male. 67 patients (53.2%) were aged <60 years, and 59 patients (46.8%) were aged ≥60 years. 52.4% of patients smoked. 82 patients (65.1%) were clinically stage IIIA, and 44 patients (34.9%) were stage IIIB. The majority of patients (98, 77.8%) recorded a KPS score of 100, with the remaining 28 (22.2%) scoring between 80 and 90. Of the 72 patients (57.1%), the pathological type was adenocarcinoma, and 54 patients (42.9%) were squamous cell carcinoma. 56 patients (44.4%) underwent open thoracotomy, and 70 patients (55.6%) underwent VAST surgery. 59 patients (46.8%) received neoadjuvant chemotherapy with paclitaxel+ platinum-based drugs, 30 patients (23.8%) received pemetrexed+ platinum-based drugs, and 37 patients (29.4%) received gemcitabine+ platinum-based drugs. 62 patients (49.2%) experienced postoperative complications.

### Relationship between mNPS and clinicopathological features

Based on the mNPS criteria, Group 0 (0 points) comprised 20 patients (15.9%); Group 1 (1–2 points) comprised 85 patients (67.5%); and Group 2 (3–4 points) comprised 21 patients (16.7%). MNPS showed no statistically significant association with any clinicopathological characteristic (all P > 0.05) (**Table 1**).

**Table 1:** Relationship between mNPS and clinicopathological characteristics in patients with locally advanced NSCLC.

Clinical characteristics	MNPS			*P value*
	Number of cases	Group 0	Group 1	Group 2
Gender				0.322
Female	66 (52.4%)	9 (45.0%)	43 (50.6%)	14 (66.7%)
Male	60 (47.6%)	11 (55.0%)	42 (49.4%)	7 (33.3%)
Age (years)				0.484
<60	67 (53.2%)	13 (65.0%)	44 (51.8%)	10 (47.6%)
≥60	59 (46.8%)	7 (35.0%)	41 (48.2%)	11 (52.4%)
Smoking				0.234
No	60 (47.6%)	13 (65.0%)	38 (44.7%)	9 (42.9%)
Yes	66 (52.4%)	7 (35.0%)	47 (55.3%)	12 (57.1%)
Clinical stage				0.397
IIIA	82 (65.1%)	14 (70.0%)	57 (67.1%)	11 (52.4%)
IIIB	44 (34.9%)	6 (30.0%)	28 (32.9%)	10 (47.6%)
KPS score				0.081
100	98 (77.8%)	19 (95.0%)	65 (76.5%)	14 (66.7%)
80-90	28 (22.2%)	1 (5.0%)	20 (23.5%)	7 (33.3%)
Pathological type				0.559
Adenocarcinoma	72 (57.1%)	12 (60.0%)	46 (54.1%)	14 (66.7%)
Squamous cell carcinoma	54 (42.9%)	8 (40.0%)	39 (45.9%)	7 (33.3%)
Tumor location				0.096
Left lung	96 (76.2%)	17 (85.0%)	60 (70.6%)	19 (90.5%)
Right lung	30 (23.8%)	3 (15.0%)	25 (29.4%)	2 (9.5%)
Degree of differentiation				0.773
High/Medium	54 (42.9%)	10 (50.0%)	35 (41.2%)	9 (42.9%)
Low	72 (57.1%)	10 (50.0%)	50 (58.8%)	12 (57.1%)
Surgical method				0.237
Open chest	56 (44.4%)	6 (30.0%)	42 (49.4%)	8 (38.1%)
VAST	70 (55.6%)	14 (70.0%)	43 (50.6%)	13 (61.9%)
Chemotherapy regimen				0.738
Paclitaxel combined with platinum	59 (46.8%)	12 (60.0%)	38 (44.7%)	9 (42.9%)
Pemetrexed combined with platinum	30 (23.8%)	4 (20.0%)	20 (23.5%)	6 (28.6%)
Gemcitabine combined with platinum	37 (29.4%)	4 (20.0%)	27 (31.8%)	6 (28.6%)
Postoperative complications				0.112
Yes	62 (49.2%)	6 (30.0%)	43 (50.6%)	13 (61.9%)
No	64 (50.8%)	14 (70.0%)	42 (49.4%)	8 (38.1%)
CEA level				0.538
Normal	66 (52.4%)	12 (60.0%)	45 (52.9%)	9 (42.9%)
Abnormal	60 (47.6%)	8 (40.0%)	40 (47.1%)	12 (57.1%)

### Survival difference analysis

**Figure 3:**
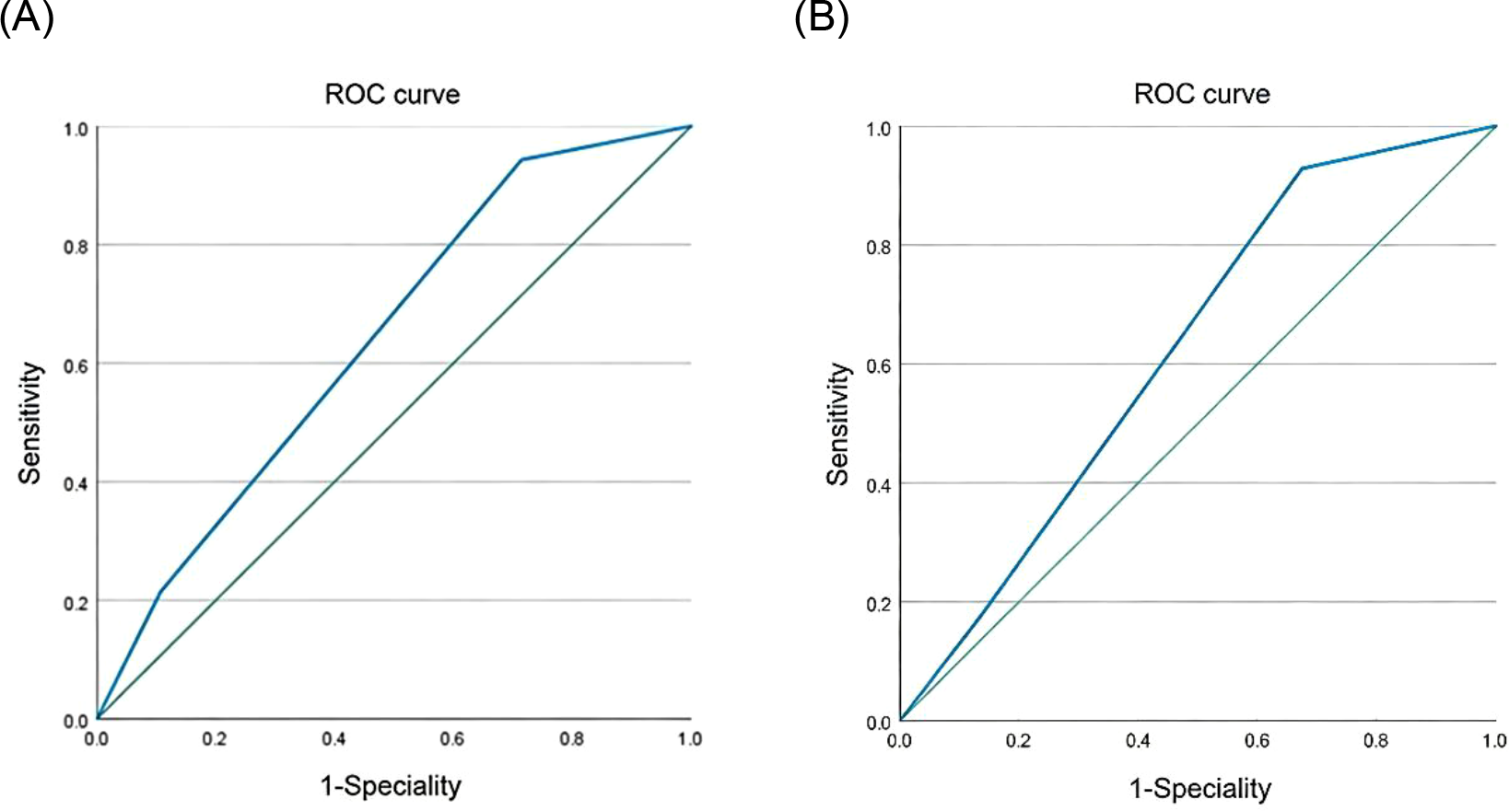
ROC curves of mNPS for predicting OS and PFS. **(A)** The ROC curve of the mNPS predicting OS in patients with locally advanced NSCLC, area under the curve AUC = 0.640. **(B)** The ROC curve of the mNPS predicted PFS in patients with locally advanced NSCLC, and the area under the curve AUC = 0.623.

**Figure 4:**
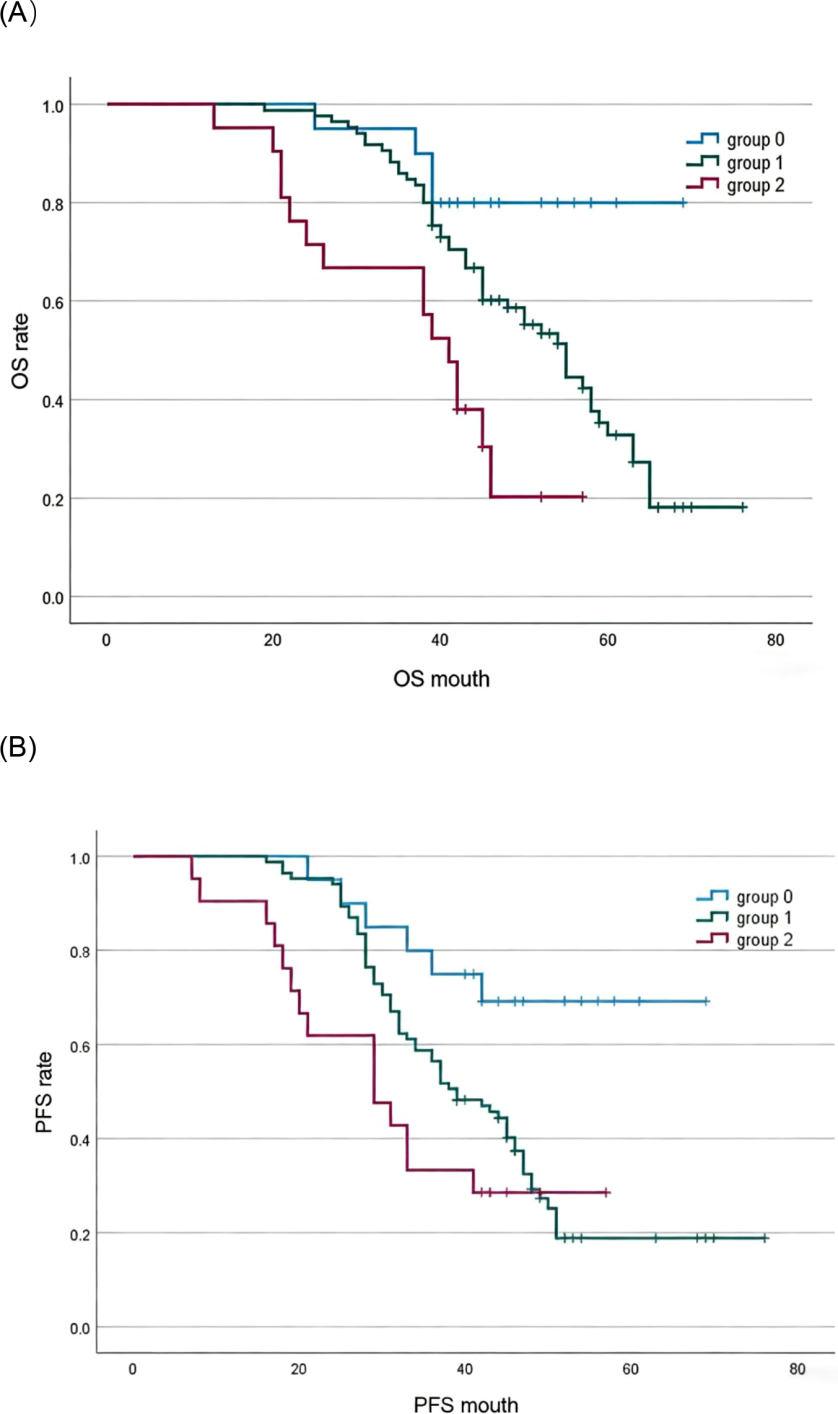
Survival curves for three Groups. **(A)** Patients in Group 0 had better OS compared with patients in Group 2 and Group 1 (Group 2 vs Group 0, P = 0.001, Group 1 vs Group 0, P = 0.038). **(B)** Patients in Group 0 had better PFS compared with patients in Group 2 and Group 1 (Group 2 vs Group 0, P = 0.004, Group 1 vs Group 0, P = 0.005). Higher mNPS correlated with worse survival.

The predictive performance of mNPS, albumin, cholesterol, LMR, and NLR was assessed via ROC curve analysis. **Table 2** lists the area under the curve (AUC) for mNPS, albumin, cholesterol, NLR, and LMR. The results indicate that mNPS had greater predictive value for OS (AUC = 0.640, P = 0.007) and PFS (AUC = 0.623, P = 0.024) than albumin, cholesterol, NLR, and LMR. The ROC curves clearly demonstrated the predictive ability of mNPS (**Figure 3**). **Figure 4** shows the OS and PFS survival curves for the three Groups. For all enrolled patients, the OS and PFS of Group 0 were superior to those of Groups 1 and 2 (OS: Group 1 vs Group 0, P = 0.038; Group 2 vs Group 0, P = 0.001; PFS: Group 1 vs Group 0, P = 0.005; Group 2 vs Group 0, P = 0.004).

**Table 2:** MNPS ROC curve analysis of blood indicators on OS and PFS.

Indicators	OS			PFS		
	AUC	95%CI	*P value*	AUC	95%CI	*P value*
ALB	0.552	0.451-0.653	0.319	0.535	0.430-0.641	0.518
CHOL	0.561	0.461-0.661	0.243	0.543	0.439-0.647	0.430
NLR	0.596	0.496-0.697	0.063	0.563	0.457-0.669	0.247
LMR	0.600	0.500-0.700	0.054	0.574	0.469-0.680	0.173
MNPS	0.640	0.542-0.738	0.007	0.623	0.514-0.732	0.024

### Single-factor and multi-factor analysis

**Table 3** presented the results of the univariate Cox proportional hazards regression analysis for clinicopathological features, mNPS, and their association with OS and PFS. Univariate analysis showed that surgical method (P = 0.042), clinical stage (P = 0.004), and KPS score (P = 0.003) were significantly associated with OS. Conversely, smoking history (P = 0.744), gender (P = 0.506), age (P = 0.460), tumor location (P = 0.419), pathological type (P = 0.969), differentiation degree (P = 0.323), CEA level (P = 0.806), and mNPS (Group 1 vs Group 0, P = 0.051; Group 2 vs Group 0, P = 0.001) were not statistically associated with OS. KPS score (P = 0.002), clinical stage (P = 0.005), and mNPS (Group 1 vs Group 0, P = 0.010; Group 2 vs Group 0, P = 0.002) were significantly associated with PFS. Other clinicopathological factors were not statistically significant with PFS. Indicators significant in the univariate analysis of PFS were included in the multivariate analysis. The multivariate analysis results showed that mNPS score (Group 1 vs Group 0, P = 0.038; Group 2 vs Group 0, P = 0.008), clinical stage (P = 0.022), and KPS score (P = 0.017) were independent prognostic factors for PFS (**Table 4**).

**Table 3:** Univariate analysis of OS and PFS in patients with locally advanced NSCLC.

Clinical characteristics	OS		PFS	
	HR (95% CI)	*P value*	HR (95% CI)	*P value*
Gender				
Female	1.000		1.000	
Male	0.852 (0.531-1.367)	0.506	0.987 (0.641-1.522)	0.954
Age (years)				
<60	1.000		1.000	
≥60	1.194 (0.746-1.911)	0.460	1.427 (0.926-2.201)	0.107
Smoking				
No	1.000		1.000	
Yes	0.925 (0.578-1.479)	0.744	0.887 (0.576-1.367)	0.588
Clinical stage				
IIIA	1.000		1.000	
IIIB	1.984 (1.240-3.174)	0.004	1.888 (1.217-2.929)	0.005
KPS score				
100	1.000		1.000	
80-90	2.112 (1.281-3.482)	0.003	2.098 (1.308-3.365)	0.002
Pathological type				
Adenocarcinoma	1.000		1.000	
Squamous cell carcinoma	1.009 (0.628-1.623)	0.969	1.212 (1.785-1.869)	0.386
Tumor location				
Left lung	1.000		1.000	
Right lung	0.794 (0.453-1.390)	0.419	1.561 (0.985-2.472)	0.058
Degree of differentiation				
High/Medium	1.000		1.000	
Low	1.273 (0.789-2.054)	0.323	0.949 (0.616-1.463)	0.813
Surgical method				
Open chest	1.000		1.000	
VAST	0.614 (0.383-0.983)	0.042	0.676 (0.439-1.041)	0.076
Chemotherapy regimen				
Paclitaxel combined with platinum	1.000		1.000	
Pemetrexed combined with platinum	0.932 (0.500-1.738)	0.825	0.929 (0.530-1.627)	0.796
Gemcitabine combined with platinum	1.007 (0.586-1.732)	0.979	1.312 (0.800-2.152)	0.283
Postoperative complications				
Yes	1.000		1.000	
No	0.739 (0.461-1.184)	0.209	0.842 (0.547-1.295)	0.434
CEA level				
Normal	1.000		1.000	
Abnormal	1.061 (0.663-1.697)	0.806	1.004 (0.652-1.547)	0.985
MNPS						
Group 0	1.000		1.000	
Group 1	2.761(0.996-7.652)	0.051	3.006(1.299-6.957)	0.010
Group 2	6.570(2.167-19.921)	0.001	4.684(1.795-12.031)	0.002

**Table 4:** Multivariate analysis of PFS in patients with locally advanced NSCLC.

	HR (95%CI)	*P value*
Clinical stage		
IIIA	1.000	
IIIB	1.681 (1.076-2.625)	0.022
KPS score		
100	1.000	
80-90	1.801 (1.113-2.915)	0.017
MNPS		
Group 0	1.000	
Group 1	2.465 (1.051-5.784)	0.038
Group 2	3.687 (1.403-9.690)	0.008

## Discussion

The tumor microenvironment, as one of the hallmarks of cancer, is the internal environment for the generation and survival of tumor cells, which includes immune and inflammatory cells (24–26). Tumors weaken anti-tumor immune responses through the tumor microenvironment, thereby maintaining proliferation, evading apoptosis, and preserving inflammatory environments and angiogenesis (26, 27). Therefore, tumor prognosis is closely related to inflammation and immunity (28, 29). In addition, nutritional status also affects tolerance to the drugs used in neoadjuvant therapy and postoperative recovery (30, 31). Studies have validated the prognostic value of inflammatory, immunological, and nutritional indicators in cancer (32–34). Research has further extended to composite scores that integrate these markers, demonstrating utility beyond that of individual utility (35, 36). The NPS is composed of albumin, cholesterol, LMR, and NLR (37). It has been confirmed to have prognostic value for multiple tumors (38–42). Previous research has shown that NPS demonstrates superior prognostic performance for resected locally advanced NSCLC patients undergoing neoadjuvant chemotherapy and surgery (20, 43). Based on the cohort of this study, the cut-off values of the four indicators were used to construct the mNPS. This study aimed to evaluate the prognostic significance of mNPS in locally advanced NSCLC. We found that mNPS was an independent prognostic factor for PFS in patients with locally advanced non-small cell lung cancer undergoing surgery after neoadjuvant chemotherapy, although it was not an independent prognostic factor for OS. The observed significance of mNPS for PFS but not OS may be attributed to several factors, including limited sample size for long-term survival events, heterogeneity in postoperative systemic therapies, and the possibility that inflammatory–nutritional influences are more pronounced in earlier disease progression rather than long-term overall survival. Although the AUC value of mNPS(0.640 for OS, 0.623 for PFS) had relatively moderate statistical significance, it was superior to a single indicator such as albumin, cholesterol, NLR, and LMR. Therefore, mNPS still has a relatively high research prospect.

## Limitations

This research has certain limitations. This study is a retrospective one, a small-sample study from a single center, and the results are greatly influenced by the sample size. Another limitation is the difference in treatment methods. All the patients with locally advanced NSCLC included in this study received neoadjuvant chemotherapy and surgical treatment, but there were differences in postoperative treatment methods. To some extent, it might affect the prediction of survival by mNPS.

## Conclusion

Although mNPS was an independent prognostic factor for PFS in patients with locally advanced non-small cell lung cancer undergoing surgery after neoadjuvant chemotherapy, it had no statistical significance for OS. In comparison, NPS demonstrated superior prognostic performance for resected locally advanced NSCLC patients undergoing neoadjuvant chemotherapy and surgery.

## Data Availability

The raw data supporting the conclusions of this article will be made available by the authors, without undue reservation.
